# Perspectives on the Efficacy of Benralizumab for Treatment of Eosinophilic Granulomatosis With Polyangiitis

**DOI:** 10.3389/fphar.2022.865318

**Published:** 2022-03-10

**Authors:** Yasuhiko Koga, Haruka Aoki-Saito, Yosuke Kamide, Makiko Sato, Hiroaki Tsurumaki, Masakiyo Yatomi, Tamotsu Ishizuka, Takeshi Hisada

**Affiliations:** ^1^ Department of Respiratory Medicine, Gunma University Graduate School of Medicine, Maebashi, Japan; ^2^ Clinical Research Center for Allergy and Rheumatology, Sagamihara National Hospital, Sagamihara, Japan; ^3^ Third Department of Internal Medicine, Faculty of Medical Sciences, University of Fukui, Fukui, Japan; ^4^ Gunma University Graduate School of Health Sciences, Maebashi, Japan

**Keywords:** asthma, eosinophilic granulomatosis with polyangiitis, benralizumab, mononeuritis multiplex, neuropathy, cardiomyopathy, mepolizumab

## Abstract

Two types of interleukin (IL)-5 antibody biologics, anti-IL-5 antibodies (mepolizumab) and anti-IL-5α receptor antibodies (benralizumab), are indicated for severe asthma. While high-dose mepolizumab is also indicated for EGPA, benralizumab is indicated only for severe asthma. Benralizumab is characterized by antibody-dependent cell-mediated cytotoxicity activity, giving them specific and rapid anti-IL-5α receptor binding abilities and the ability to target a high number of eosinophils in tissues as well as peripheral blood. Recently, reports on the efficacy of benralizumab as a treatment for EGPA have been published, along with reports on some cases that are difficult to treat with existing oral corticosteroids and mepolizumab. Therefore, we focus on the perspective of the efficacy and safety of benralizumab as a treatment for EGPA patients with steroid dependence in this review. A total of 41 patients with EGPA were treated with benralizumab. After the introduction of benralizumab, oral corticosteroids could be reduced to 10 mg/day or less in all cases and to less than 5 mg/day in 80% or more of the cases. Discontinuation of oral corticosteroids was achieved in more than 40% of patients with EGPA. Benralizumab was effective in patients with mepolizumab-refractory EGPA and intractable cardiac and neuropathy complications. Efficient elimination of eosinophils is expected to improve the remission rate of EGPA with benralizumab treatment. Although the total number of patients was small, benralizumab was safe and tolerable in a wide range of age groups, suggesting efficacy in severe cases with EGPA.

## Introduction

Eosinophilic granulomatosis with polyangiitis (EGPA) is an intractable disease of unknown etiology, and classical treatment with oral corticosteroids (OCS) and immunosuppressants are the mainstream treatment for EGPA (Furuta et al., 2019). However, EGPA treatment is difficult and has many problems including frequent recurrences, adverse events caused by long-term treatment, severe cardiac and neurological involvement, and steroid-refractory cases (Greco et al., 2015). In recent years, interleukin (IL)-5-targeted anti-IL-5 antibody (Ab) therapy has been used worldwide, including Europe and Japan since its approval by the United States Food and Drug Administration (FDA). Treatments targeting IL-5 not only improved the remission rate of EGPA but also reduced the effective dose of OCS, resulting in many benefits for patients with EGPA. Nevertheless, there are still reports of refractory EGPA, and a preferable EGPA treatment has not yet been developed. There are two types of biologics that target IL-5: mepolizumab, an anti-IL-5 Ab, and benralizumab, an anti-IL-5 receptor *α* Ab. The latter is characterized by the ability to rapidly remove eosinophils in the tissues and blood by antibody-dependent cell-mediated cytotoxicity (ADCC) activity mediated by natural killer (NK) cells (Nagase et al., 2020). To gather translational data on benralizumab in the treatment for EGPA, a systemic literature search using the keyword “benralizumab” was performed using the PubMed and Web of Science on January 28 in 2022. We extracted publications of benralizumab on the mechanism of eosinophil removal and the treatment of EGPA. In this review, we summarize the previous literature published on the efficacy of benralizumab in the treatment of EGPA. Benralizumab with ADCC activity may be considered as a future treatment option for EGPA.

## Clinical Efficacy of Benralizumab for Eosinophil Depletion

The etiology of EGPA is largely unclear, which is probably due to complicated interactions, with genetic and environmental factors causing the inflammatory response, the main agonists of which are eosinophils and T and B lymphocytes (Cottin, 2016). Activated T lymphocytes with the T helper type2 phenotype activate, matures, and help to survive eosinophils by secreting cytokines such as IL-4, IL-5, and IL-13. In particular, the serum IL-5 level is increased in patients with active EGPA (Moosig et al., 2011; Vaglio et al., 2012), and inhibition of this increased level is thought to be a potential therapeutic target. Eosinophils are present primarily in the tissues rather than in the blood. The life cycle of eosinophils consists of three stages: bone marrow, blood, and tissue. When eosinophils enter the blood from the bone marrow, their half-life span is as short as 8–18 h (Nagase et al., 2020). After circulating in the blood, eosinophils migrate to tissues, extending their lifespan to 2–5 days. The eosinophil blood:tissue ratio in humans is approximately 1.14:100. Eosinophils are distributed in tissues *via* systemic circulation (Laviolette et al., 2013).

**FIGURE 1:**
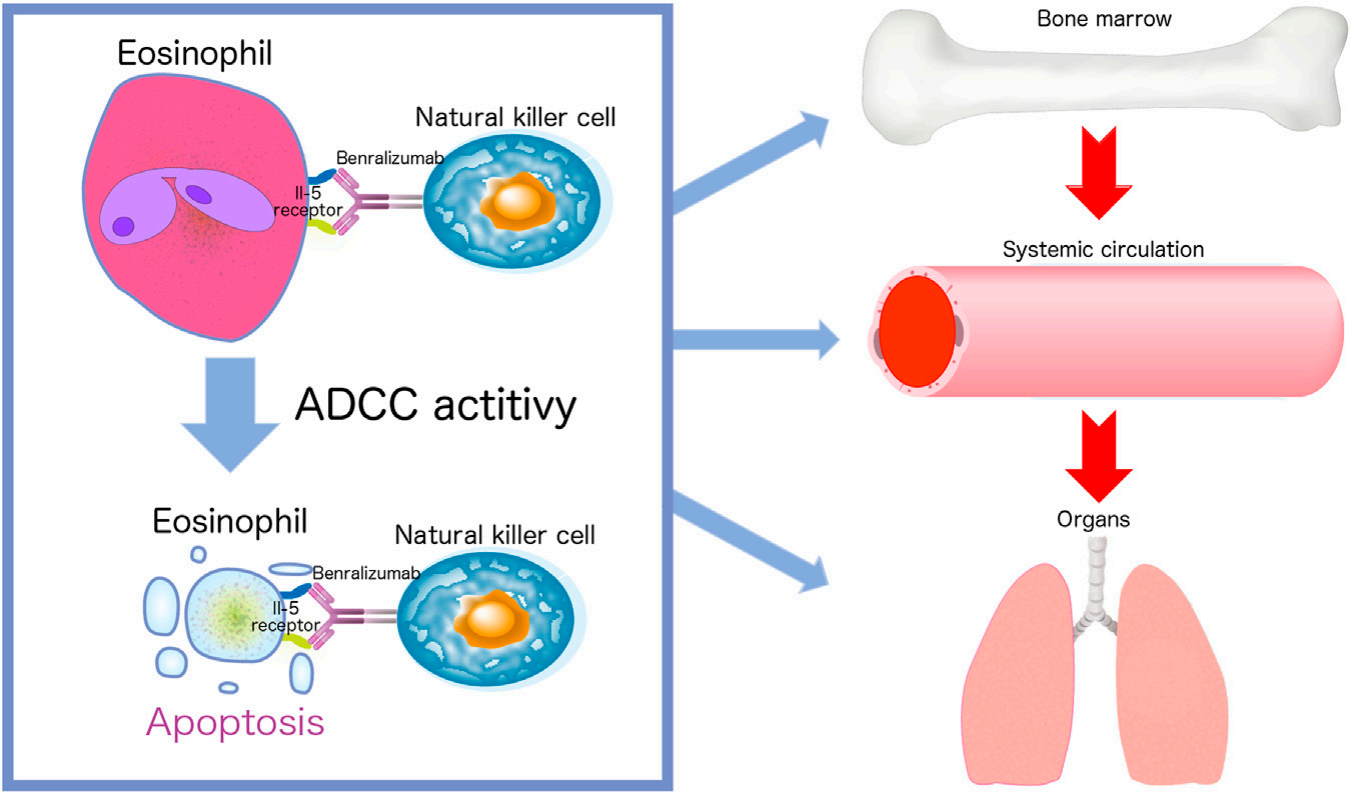
Afucosylated benralizumab enables natural killer cells to recognize benralizumab bound to eosinophils. The life cycle of eosinophils consists of three stages: bone marrow, blood, and tissue. Benralizumab causes eosinophil apoptosis due to its high affinity for the FcγRIII receptors through antibody-dependent cell-mediated cytotoxicity (ADCC). Benralizumab reduces circulating and tissue resident eosinophils and inhibits eosinophil differentiation and maturation in the bone marrow.

Benralizumab has a very specific mechanism of action compared with that of mepolizumab because of its ability to reduce both circulating and resident eosinophil counts by enhancing ADCC. Benralizumab is an afucosylated monoclonal antibody that lacks fucose sugar in a crystallizable antibody fragment. This modification of human IgG1 results in a 5- to 50-fold higher affinity for human FcγRIIIa receptors expressed on NK cells, macrophages, and neutrophils (Shinkawa et al., 2003). Removal of fucose from the Fc region of benralizumab enables NK cells to recognize benralizumab bound to eosinophils. Benralizumab causes eosinophil apoptosis due to its high affinity for the FcγRIII receptors through ADCC (Figure 1) (Shinkawa et al., 2003; Kolbeck et al., 2010). The afucosylated crystallizable antibody fragments reduce circulating and resident eosinophils, and antigen-binding fragments of benralizumab inhibit eosinophil differentiation and maturation in the bone marrow (Hassani and Koenderman, 2018). Dagher et al. found that activated NK cells subsequently promote tumor necrosis factor expression by macrophages *via* the secretion of interferon (IFN)-γ. These results suggest that this may contribute to the potent antieosinophilic activity of benralizumab *in vivo* (Dagher et al., 2021). Recent findings also indicate that benralizumab treatment restores the expression levels of the key molecules involved in steroid sensitivity (Hirai et al., 2021). These mechanisms of action are unique to benralizumab. In addition, the afucosylated form improves the interaction between benralizumab and the IL-5 receptor, thereby increasing ADCC (Menzella et al., 2016), and thus reducing the number of circulating eosinophils by 90–100% and the number of resident eosinophils in various tissues, such as the lung and the bone marrow (Menzella et al., 2019) (Figure 1). Almost no eosinophils and basophils can be detected in the blood, and eosinophil precursors in the bone marrow show a reduction of more than 80% (Menzella et al., 2016). Benralizumab can reduce baseline eosinophils by 89.8% in the airway mucosa, 89.9% in the sputum, and 100% in the bone marrow (Laviolette et al., 2013). Furthermore, benralizumab does not cause degranulation through eosinophil cell lysis that in turn releases harmful proteins, such as eosinophil cationic proteins and eosinophil-derived neurotoxins through ADCC (Hassani and Koenderman, 2018). It significantly reduces the levels of IL-5 receptor-carrying cells and ultimately stops the vicious cycle of tissue damage (Faverio et al., 2018; Hassani and Koenderman, 2018). These features may be beneficial in the treatment of EGPA using benralizumab.

## Clinical Case Studies of Benralizumab as Treatment for EGPA

Recently, the optional treatment using benralizumab with its steroid-sparing effect on EGPA has been promising. An open-label prospective pilot study to evaluate the safety and efficacy of benralizumab was performed, enrolling 10 patients with EGPA (Guntur et al., 2021). On an average, 3 years since the diagnosis of EGPA the positive rate for antineutrophil cytoplasmic antibody (ANCA) was 30%. During the treatment period, although three patients were unable to reduce the OCS dose from baseline, including two patients who were positive for ANCA, half of the patients were able to successfully discontinue OCS. The reduction in OCS dosage was 15 mg at the baseline to 2 mg at the end of benralizumab treatment. In the treatment-free phase following five injections of 30 mg benralizumab, frequent EGPA exacerbations were observed, indicating a lack of sustained treatment effect following discontinuing of benralizumab. Although three patients were ANCA-positive at baseline, two patients converted to ANCA-negative by the end of the study, while one patient sustained serine proteinase 3 ANCA positivity throughout the entire study (Guntur et al., 2021).

Two case studies have investigated the efficacy of benralizumab in patients with EGPA including mepolizumab-refractory patients. Padoan et al. analyzed benralizumab treatment in five patients with EGPA over 4 months. There was 1 patient with myeloperoxidase (MPO)-ANCA and 1 patient with neuropathy, and mepolizumab was not effective in 3 of 5 of the patients in the 6 months prior to benralizumab treatment (all 3 of whom were MPO-ANCA-negative). After 24 weeks of treatment (5 injections) with benralizumab, the OCS dose could be reduced from an average of 12.5 to 0 mg, and 3 of the patients were able to discontinue OCS therapy and had a complete depletion of peripheral eosinophils (Padoan et al., 2020).

In another case study, 11 patients with refractory EGPA underwent a 24-weeks benralizumab treatment, nine of whom completed treatment in 48 weeks. There were four patients with ANCA-positive EGPA, 2 with cardiac involvement, and 1 with neurological involvement. Mepolizumab failed in 3 of the 11 patients, and in 1 both mepolizumab and reslizumab were unsuccessful. The median OCS therapy duration was reduced from 15 mg (10–20 mg) to 5 mg (5–10 mg) at 24 weeks and to 5 mg (1–6.5 mg) at 48 weeks, and there was an overall reduction in peripheral eosinophils. Improvement in the Birmingham Vasculitis Activity Score (BVAS) and Asthma Control Questionnaire score were obtained after 24 weeks of benralizumab treatment (Table 1) (Nanzer et al., 2020).

**TABLE 1:** Case series of the efficacy of benralizumab for treatment of eosinophilic granulomatosis with polyangiitis.

Author, Year	Number of patients	Age/Gender	Previous therapy of mepolizumab (300 mg)	ANCA positivity (rate)	OCS dose (mg) (pre)	OSC dose (mg) (post)	OCS withdrarw	ACQ (Pre/Post)	BVAS (Pre/Post)	Treatment periods (week)	Cardiac improvement	Neuropathy improvement
Guntur et al. (2021)	10	47 (mean)	0	3/10, 30% (2/MPO; 1/PR3)	11.6 (6.4–20.5)	5.3 (2.6–9.85)	5/10 (50%)	2.5 (1.7–3.5)/2.7 (1.9–3.8)	10/no significant difference	24	none	no response (1 patient)
Pandoan et al. (2020)	5	42 (mean)	3/5 (60%)	1/5, (20%) MPO (1)	12.5 (11.25–15)	0 (0–3.12)	3/5 (60%)	19 (17–21)/4 (0–7)	4 (3–4)/0 (0–0)	24	none	1 case
Nanzer et al. (2020)	11	50 (mean)	3/11 (27.3%)	4/11, (36%)	15 (10–20)	5 (5–10)	2/9 (22.2%)	2.13 (1.15–3.11)/1.73 (0.16–3.30)	7.91 (4.64–11.18)/3.45 (0.93–5.97)	24 (*n* = 11)48 (*n* = 9)	2 cases	1 case
Takenaka et al. (2019)	1	59/M	(−)	(+) MPO	6	5	no	N.D.	N.D.	16	N.A.	N.A.
Coppola, (2020)	1	63/F	(−)	(+) PR3	25	5	no	N.D.	N.D.	12	N.A.	(+)
Colantuono et al. (2020)	1	19/M	(−)	(−)	N.D.	5	no	N.D.	N.D.	56	(+)	(+)
Lee and Hojjati, (2020)	1	71/M	(−)	(−)	50	0	yes	N.D.	17/11 (after 2 months)	22	(−)	(+)
Carrillo-Martin, (2020)	1	23/M	(−)	(−)	40	10	no	N.D.	N.D.	55	N.A.	N.A.
Chica-Guzman et al. (2020)	1	53/M	(+) 100 mg dosage	N.D.	N.D.	0	yes	N.D.	N.D.	26	N.D.	N.D.
Martinez-Rivera, (2021)	1	57/M	(−)	(+) MPO	80 (mPSL)	5	no	N.D.	N.D.	3	(+)	N.A.
Hinds et al. (2021)	1	14/F	(−)	(+) MPO	1000, mPSL	N.D.	N.D.	N.D.	N.D.	34	(+)	N.A.
〃	1	7/F	(−)	(−)	2 mg/kg/day	0	yes	N.D.	N.D.	30	N.A.	N.A.
〃	1	15/F	(−)	(+) MPO	1000, mPSL	N.D.	N.D.	N.D.	N.D.	26	N.A.	N.A.
Bormioli et al. (2021)	1	53/M	(+)	N.D.	12.5	7.5	no	3.8/1.6	8/1	52	(+)	N.A.
Kolios et al. (2021)	1	58/M	(−)	(+)	0 → 1000, mPSL	0	yes	N.D.	N.D.	104	(+)	N.A.
Lim et al. (2021)	1	31/M	(−)	(+) MPO	<37.5 mg	<10 mg	no	N.D.	N.D.	44	N.A.	N.A.
Menzella et al. (2021)	1	57/M	(+) 100 mg dosage	(+) MPO	25 mg	0 (6M)	yes	3/1	N.D.	52	N.A.	N.A.
Miyata et al. (2021)	1	38/M	(−)	(+) MPO	30 mg	4 mg	no	N.D.	16/1	104	N.A.	(+)
Total, Average	41	—	9/41 (22.0%)	17/39, 43.6%	0–1000 mg	0–10 mg	15/37, 40.5%	—	—	34.7 ± 2.75	7/7	6/7

ANCA,antineutrophil cytoplasmic antibody;OCS, oral corticosteroid; ACQ, asthma control questionnaire; BVAS, birmingham vasculitis activity score; M, male; F, female, MPO; myeloperoxidase; PR3, serine proteinase3; mPSL, methylpredonisolone; N.D., not described; N.A., not applicable.

In these case studies, benralizumab (30 mg) was administered by subcutaneous injections every 8 weeks, with the first three doses administered every 4 weeks.

An open-label phase 2 clinical trial evaluating the safety and efficacy of benralizumab in patients with EGPA treated with OCS and/or immunosuppressants is underway (BITE) (NCT03010436). Another randomized, double-blind phase 3 clinical trial has been evaluating the efficacy and safety of benralizumab (30 mg) compared to mepolizumab (300 mg) in patients with EGPA is ongoing (MANDARA) (NCT04157348).

## Case Reports of Benralizumab as Treatment for EGPA

Twelve case reports and one pediatric case series have been reported for benralizumab use in patients with EGPA. As shown in Table 1, the average age of the patients in these case reports was 41.2 ± 21.17 years, and 11/15 of the patients were men. A minimum of 3 weeks of treatment was reported, and the average observation period for benralizumab treatment was 38.9 ± 24.8 weeks. Three patients had prior treatment with mepolizumab, two of whom had received a lower dose of mepolizumab–100 mg–due to local disapproval. The proportion of patients with ANCA-positive was as high as 9/13 (69%), and the proportion of patients in whom OCS could be discontinued during the observation period was 3/12 (25%). The initial dosage of OSC varied from 0 to 80 mg/day, except for two patients, who received 1 g/day of steroid pulse therapy. After the introduction of benralizumab, OCS could be reduced to 10 mg/day or less in all patients and to less than 5 mg/day in 80% or more of the patients. These case studies included a high proportion of ANCA-positive EGPA cases, and, four of the ANCA-positive patients showed a decreasing trend in MPO-ANCA during treatment with benralizumab (Takenaka et al., 2019; Guntur et al., 2021; Kolios et al., 2021).

In children with EGPA, the specific diagnosis of each case remains difficult due to their being a lack of child-specific diagnostic criteria for EGPA. As a result, treating children with EGPA can be challenging. In four out of fifteen patients in these case series under the age of 20 years, the efficacy, safety, and tolerability of benralizumab for more than 6 months were confirmed. Since benralizumab was approved by the FDA in 2017 for severe eosinophilic asthma in patients aged 12 years and older, benralizumab may become a potential therapeutic option for refractory EGPA in children.

Case studies and case reports suggest that benralizumab has a steroid-sparing effect on EGPA. Distinct from these reports, two cases have been reported in which discontinuation of OCS after benralizumab treatment results in a worsening of EGPA (Hocevar et al., 2020). In addition, there has been a case report of a patient with severe asthma developing EGPA after reducing the dose of OCS to 5 mg/day during benralizumab treatment (Lim et al., 2021). It may be necessary to pay attention to the exacerbation of EGPA after OCS tapering or discontinuation during benralizumab treatment.

## Efficacy of Benralizumab on Mepolizumab-Refractory EGPA

The efficacy of benralizumab in reducing the number of eosinophils in the blood and lung tissues appears to be more pronounced than that of mepolizumab (Flood-Page et al., 2003; Laviolette et al., 2013). In a phase 2 trial evaluating the effectiveness of benralizumab in hypereosinophilic syndrome (HES), three patients had prior treatment with mepolizumab, and one achieved remission (Kuang et al., 2019).

In two case studies, benralizumab was introduced after pretreatment with mepolizumab in 3/5 (60%) patients in one and 3/11 (27.3%) patients in the other case study. Padoan et al. reported three patients refractory to mepolizumab treated for 16, 19, and 18 months (one patient was administered 100 mg for 5 months followed by 300 mg). All patients were female and ANCA-negative and were administered PSL 10 mg/day, 5 mg/day, and 15 mg/day, respectively. Benralizumab treatment resulted in a complete depletion of peripheral eosinophils in all three patients (patient 1: 1000 to 0/μL, patient 2: 1200 to 0/μL, patient 3: 150 to 0/μL) (Padoan et al., 2020). Nanzer et al. reported the efficacy of benralizumab in three patients with EGPA who had previously experienced treatment failure with mepolizumab, and one patient had experienced treatment failure with both reslizumab and mepolizumab. Peripheral eosinophils were completely depleted after 24 weeks of treatment (Nanzer et al., 2020).

Interestingly, Bormioli et al. reported a biopsy case demonstrating the disappearance of eosinophils in tissues after benralizumab treatment. Transbronchial lung biopsy (TBLB) was performed during treatment with mepolizumab (300 mg) followed by benralizumab (30 mg). During treatment with mepolizumab, asthma worsened, and bronchoalveolar lavage was performed. Although the proportion of eosinophils in the bronchoalveolar lavage fluid was 0%, TBLB revealed submucosal IL-5α-positive eosinophil infiltration and vasculitis findings. Thereafter, TBLB during a subsequent 1-year benralizumab treatment showed no findings of IL-5α-positive eosinophil infiltration or vasculitis. Moreover, in this case, OCS was reduced from 12.5 to 7.5 mg/day, the forced expiratory volume in the first second was improved from 2.25 to 2.73 L, and a significant improvement was observed in the questionnaire scores regarding asthma and rhinitis during the benralizumab treatment (Bormioli et al., 2021).

Another patient with ANCA-positive without improvement in his asthma symptoms after seven doses of mepolizumab (100 mg) was able to reduce OCS dosage from 25 to 0 mg/day after 1 year of benralizumab treatment. Respiratory symptoms progressively improved after 3 months. The peripheral blood eosinophil count and MPO-ANCA test results were negative. In this patient, due to the regional disapproval of mepolizumab for use in treating EGPA, treatment with 300 mg of mepolizumab could not be considered (Menzella et al., 2021).

In a report on a patient refractory to EGPA treated with omalizumab and mepolizumab, benralizumab was introduced after 2 years of omalizumab administration followed by 6 months of mepolizumab (100 mg/day), and OCS was discontinued and stable for 6 months during the benralizumab treatment (Chica-Guzman et al., 2020).

Recently, a patient with EGPA with a suboptimal response to benralizumab, as indicated by a lack of eosinophil depletion, was reported. Impaired NK cell activity may have been the cause, suggesting a lack of NK cell-releasing IFN-γ in macrophage-mediated eosinophil apoptosis in benralizumab-refractory cases. Lack of response to benralizumab therapy may be partly because of the lack of NK activity, in addition to the development of anti-drug neutralizing antibodies (Poznanski et al., 2021).

## Efficacy of Benralizumab on Cardiac and Neurological Involvement in EGPA

Most cases of mononeuritis multiplex complicated with EGPA are refractory to OCS therapy. The efficacy of mepolizumab or benralizumab in cardiomyopathy and neuropathy has not been elucidated.

In a case report of mepolizumab, neuropathy improved 4 weeks after mepolizumab administration following methylprednisolone pulse and intravenous immunoglobulin in a patient with EGPA (Nishihara et al., 2021). A recent retrospective case study in six patients with EGPA demonstrated the efficacy of mepolizumab on neuropathy refractory to OCS with intravenous immunoglobulin over 12 weeks (Kitamura et al., 2021). Higashitani et al. reported that the combination therapy of mepolizumab with rituximab achieved remission of myocarditis with EGPA (Higashitani et al., 2022). A clinical study of mepolizumab for treating HES showed a poor response in patients with cardiac manifestations (Kuang et al., 2018). Monthly injections of benralizumab for 1 year showed clinical and hematologic responses in 74% patients with HES in a randomized clinical trial (Kuang et al., 2019).

Regarding the efficacy of benralizumab, Colantuono et al. reported that benralizumab was safe and effective against refractory cardiac and neuropathy involvement in a 19-year-old patient with EGPA. Neuropathy improved, and a colon biopsy was performed at 7 months of benralizumab therapy showed regression of the eosinophilic infiltration. The ejection fraction had increased from 40 to 60% after 2 months of benralizumab treatment and was maintained throughout the follow-up period of 12 months (Colantuono et al., 2020). In the patient with EGPA with neuropathy, 2 months after the introduction of benralizumab, the BVAS improved from 15 to 11 and OSC was discontinued. His rheumatological examination revealed improvement in neuropathy 3 months after benralizumab treatment and a stable effect for 6 months (Lee and Hojjati, 2020). Another case also demonstrated a relatively rapid improvement of mononeuritis multiplex after benralizumab treatment in 1 month (Miyata et al., 2021). Combination therapy of rituximab with benralizumab recovered pulmonary hypertension to the baseline levels (Hinds et al., 2021). In a severe case, cardiac thrombus and septic embolization by *Staphylococcus aureus* sepsis in a patient with EGPA was recovered by combination therapy of benralizumab and antibiotics with 1 g/day methylprednisolone pulse therapy tapering to 0 mg during the initial 2 months of treatment (Kolios et al., 2021).

In this review, there were 7 cases of cardiac lesions, 7 of neurological lesions, and 2 of both cardiac and neuropathic complications in patients with EGPA (Table 1). Benralizumab was effective in most of these cases, and benralizumab was also effective in two cases with both cardiac and neurological complications for more than 1 year. Colantuono et al. suggested that early intervention with anti-IL-5 treatment may be effective before irreversible myocardial damage with extensive fibrosis develops in patients with EGPA.

Biopsies performed in two case reports showed regression in eosinophil infiltration in the tissues after benralizumab treatment (Colantuono et al., 2020; Bormioli et al., 2021).

## Conclusion and Future Perspectives

In these case series, benralizumab was effective in patients with mepolizumab-refractory EGPA and in patients with intractable cardiac and neuropathy complications. The mechanisms of the effectiveness of benralizumab for mepolizumab-refractory and cardiac and neurological involvement in patients with EGPA have not yet been elucidated. One possible scenario is that benralizumab not only depletes eosinophils in the bone marrow and blood but also facilitates eosinophils in tissues to undergo ADCC-mediated apoptosis by NK cells. Efficient elimination of eosinophils, which are essential for the pathophysiology of EGPA, is expected to ultimately improve the remission rate of EGPA with benralizumab treatment. The ANCA-positive rate of successful benralizumab cases was relatively high, but the relationship between ANCA positivity and the effects of benralizumab has not been elucidated. Future clinical trials are required to assess the efficacy and safety of benralizumab treatment for refractory EGPA.
